# Potential of Fermented Food-Derived *Lactiplantibacillus* Cell-Free Supernatants to Control *Staphylococcus aureus* Growth and Biofilm Development

**DOI:** 10.3390/ijms27020760

**Published:** 2026-01-12

**Authors:** Lena Ilieva, Vesselin Baev, Mariana Marhova, Galina Yahubyan, Elena Apostolova, Mariyana Gozmanova, Velizar Gochev, Tsvetelina Paunova-Krasteva, Tsvetozara Damyanova, Sonya Kostadinova, Miroslava Gocheva, Ivan Iliev

**Affiliations:** 1 Department of Biochemistry and Microbiology, Faculty of Biology, University of Plovdiv, 24 Tsar Asen Str., 4000 Plovdiv, Bulgaria; lena.ilieva@abv.bg (L.I.); marhova@uni-plovdiv.bg (M.M.); vgochev@uni-plovdiv.bg (V.G.); skosta@uni-plovdiv.bg (S.K.); 2 Agricultural Academy, Sofia, Institute of Food Preservation and Quality-Plovdiv, 154 Vasil Aprilov Blvd., 4003 Plovdiv, Bulgaria; 3 Department of Molecular Biology, Faculty of Biology, University of Plovdiv, 24 Tsar Asen Str, 4000 Plovdiv, Bulgaria; gyahubyan@uni-plovdiv.bg (G.Y.); eapostolova@uni-plovdiv.bg (E.A.); mariank@uni-plovdiv.bg (M.G.); 4 Stephan Angeloff Institute of Microbiology, Bulgarian Academy of Sciences, Akad. G. Bonchev St. bl. 26, 1113 Sofia, Bulgaria; pauny@abv.bg (T.P.-K.); tsvetozaradamianova@gmail.com (T.D.); 5 Scientific and Research Laboratory, Centre for Research Projects and Technological Transfer, University of Food Technologies, 4000 Plovdiv, Bulgaria; m_gocheva@uft-plovdiv.bg

**Keywords:** *Lactiplantibacillus*, LAB, *Staphylococcus aureus*, bacteriocines, whole genome sequencing, ONT

## Abstract

*Staphylococcus aureus* biofilms represent a critical healthcare challenge, driving chronic infections and antimicrobial resistance. This study investigates the anti-staphylococcal efficacy of two *Lactiplantibacillus* strains isolated from traditional Bulgarian pickled vegetables (turshiya): *L. plantarum* IZITR_24 and *L. paraplantarum* IZITR_13. Combining whole genome sequencing (WGS) with functional assays, we established a robust genotype-to-phenotype framework to characterize their antimicrobial arsenal. Based on WGS, we identified conserved plantaricin (plnJK, plnEF) clusters in both isolates, with IZITR_13 additionally carrying genes for pediocin and enterolysin A—alongside the confirmed absence of virulence factors. Reconstituted lyophilized cell-free supernatants (LCFSs) were evaluated in dose–response microtiter assays to determine the minimum biofilm inhibitory concentration (MBIC) and minimum inhibitory concentration (MIC). Both strains demonstrated clear, dose-dependent inhibitory activity against the *S. aureus* growth and biofilm formation. Microscopy (SEM/CLSM) confirmed significant biofilm disruption and cell membrane permeabilization. The observed consistency between genome-inferred capacity and phenotypes highlights the strong predictive value of a genome-first screening approach for selecting bacteriocin-producing lactic acid bacteria (LAB). These findings position IZITR_24 and IZITR_13 as promising postbiotic producers with potent antibiofilm activity against *S. aureus*. By utilizing their stable postbiotic products rather than relying on live colonization, this study proposes a targeted, antibiotic-sparing strategy to combat persistent staphylococcal biofilms.

## 1. Introduction

*Staphylococcus aureus* is a significant human pathogen responsible for diseases in both community and healthcare environments, and it is closely associated with biofilm-related, chronic, and device-associated infections. Biofilm growth facilitates metabolic heterogeneity, provides protection against host immunity, and confers tolerance to standard antibiotics, all of which hinder eradication efforts and promote relapse [1,2]. At the same time, the global burden of antimicrobial resistance (AMR) continues to rise, with methicillin-resistant *Staphylococcus aureus* (MRSA) being one of the main causes of death and disease. This phenomenon shows how important non-traditional, antibiofilm treatments are [3]. Nasal carriage, which is common in about a quarter of the general population, serves as a reservoir and risk factor for subsequent infections, including surgical site and skin/soft-tissue diseases [4,5].

The formation of biofilm in *S. aureus* entails adhesion, accumulation, and maturation within an extracellular matrix, regulated by circuits such as the *agr*-quorum-sensing system and pathways governing polysaccharide intercellular adhesin (e.g., *ica*ADBC), among others [2,6,7]. These networks facilitate immune evasion, stress resilience, and dispersal mechanisms that perpetuate chronic infection [7,8]. Consequently, therapies that inhibit biofilm formation or disrupt existing biofilms—preferably without inducing additional resistance—are of paramount importance [1,2].

An emerging strategy is to use postbiotics, which are non-viable microbial preparations and their components with proven health benefits, thereby leveraging antimicrobial chemistry while avoiding the complexities of live microbe colonization at extra-intestinal sites like the anterior nares [9]. Postbiotics provide practical benefits, including stability, dose standardization, and safety. They can also be engineered or mixed to target certain pathogens and phenotypes, such as biofilms. Within lactic acid bacteria (LAB), cell-free culture supernatants (CFSs) comprise organic acids, biosurfactants, small peptides/bacteriocins, and other components that collectively inhibit growth, disrupt adhesion, and remodel biofilm architecture [10,11,12].

Research on *S. aureus* shows that LAB-derived cell-free supernatants (CFSs) can stop planktonic growth and limit biofilm formation in a dose-dependent way. This has been seen during biofilm development and, at higher concentrations, against established biofilms. Mechanistically, these postbiotic mixtures can acidify the microenvironment, disrupt quorum-sensing and exopolysaccharide pathways, and permeabilize membranes through bacteriocin-like activities, without easily promoting resistance [13,14]. These characteristics render postbiotics promising adjuncts or alternatives in antibiofilm strategies for MRSA/MSSA colonization and infection [10].

*Lactiplantibacillus* spp. are particularly appealing producers within LAB because many strains synthesize class II bacteriocins (like plantaricins PlnJK/PlnEF) that are active against *S. aureus*, including MRSA, through pore formation and cell wall damage [15,16,17]. These peptides, along with co-produced acids and surfactants, provide a logical basis to anticipate both growth inhibition and biofilm reduction by whole CFSs rather than individual purified components [15,16]. Preliminary research has indicated that lactobacilli-derived CFSs inhibit the growth of *Staphylococcus* and the formation of biofilms [18,19,20,21], and also modulate the expression of *agr*-linked virulence genes in MRSA [22].

Based on these findings, our objective was to assess the impact of complete, cell-free supernatants (CFSs) derived from *Lactiplantibacillus* strains, isolated from traditional Bulgarian fermented vegetables (turshiya), on the growth and biofilm formation of *S. aureus*. In parallel with these functional assays, we aimed to perform whole genome sequencing and in silico profiling of bacteriocin loci and probiotic/safety markers to mechanistically anchor the observed effects, verify baseline safety expectations for the producers, and benchmark genome-guided predictions against in vitro phenotypes as a resource-efficient “dry–wet” pipeline. We hypothesize that intact CFSs, which include organic acids and bacteriocin-rich fractions, will inhibit planktonic proliferation and diminish biofilm biomass and viability compared to controls, with genome features (e.g., plantaricin systems) aligning with the inhibitory profile.

## 2. Results and Discussion

### 2.1. Genomic Exploration of L. plantarum IZITR_24 and L. paraplantarum IZITR_13 Through Whole Genome Sequencing

The genome of *L. plantarum* IZTR_24 consists of a circular assembly totaling 3,310,887 base pairs (bp) with a guanine–cytosine (GC) content of 44.3%. In comparison, the genome of *L. paraplantarum* IZTR_13 is slightly larger, spanning 3,411,310 bp with a GC content of 43.6%. Both genomes exhibit a similar overall organization; *L. plantarum* IZTR_24 contains 2908 coding sequences, while *L. paraplantarum* IZTR_13 encodes 3068 coding sequences. The RNA profiles are comparable, with IZTR_24 containing 83 RNAs and IZTR_13 containing 82 RNAs. While both strains contain ribosomal and transfer RNA sets typical of the genus, a notable distinction was identified in their immune systems: CRISPR arrays were identified in *L. paraplantarum* (three loci), whereas none were reported in *L. plantarum*.

**Figure 1:**
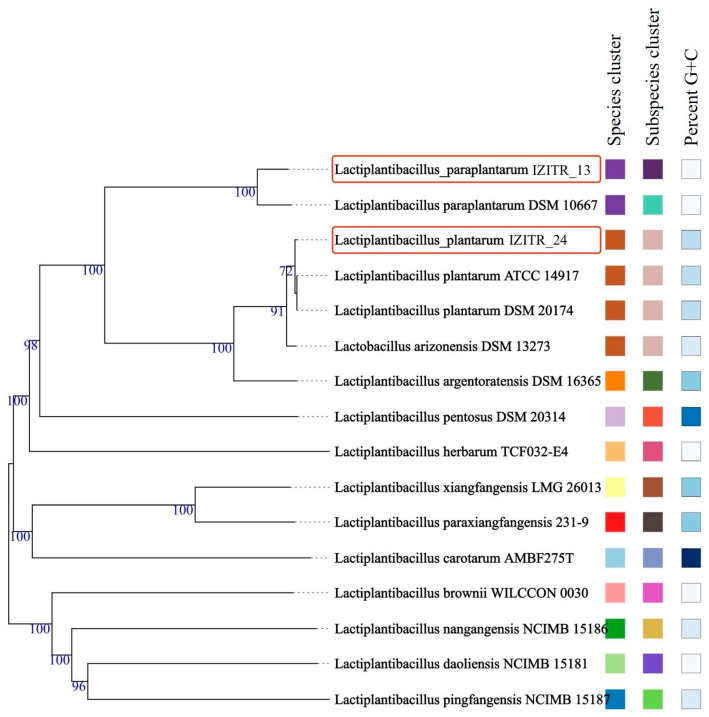
Confirmation of the *L. plantarum* IZITR_24 and *L. paraplantarum* IZITR_13 via genome-to-genome comparisons in TYGS.

To further validate their taxonomic profile, phylogenomic and multilocus sequence typing (MLST) analyses were employed. These approaches provide precise species-level discrimination and facilitate cross-study comparisons, based on the analysis of the sequences of a number of housekeeping genes [23,24]. Genome-to-genome TIGS analyses confirmed the classification, placing *L. paraplantarum* IZTR_13 and *L. plantarum* IZTR_24 into their respective species clusters and verifying the strain taxonomy (Figure 1).

In the context of carbohydrate metabolism—a critical factor for a probiotic’s ability to utilize prebiotic fibers and persist in the gut—both strains display a similar total metabolic capacity, with approximately 390 genes dedicated to carbohydrates. However, *L. plantarum* IZTR_24 exhibits a higher potential for processing complex sugars, possessing 137 genes for di- and oligosaccharides compared to 113 in IZTR_13. Specifically, IZTR_24 is notably richer in genes for beta-glucoside metabolism (41 genes) and maltose utilization (32 genes) compared to IZTR_13, which contains 30 genes for each of these categories. This suggests that IZTR_24 may be more efficient at fermenting a wider variety of plant-derived oligosaccharides, potentially offering superior symbiotic efficacy when paired with specific prebiotics. Conversely, IZTR_13 shows specific adaptations of its own, including a higher number of genes dedicated to D-tagatose and galactitol utilization.

Regarding survival in the gastrointestinal tract, which requires robust adaptation to harsh conditions, *L. paraplantarum* IZTR_13 appears to possess a more comprehensive stress response system (Table S1). The analysis identifies 73 stress response genes in IZTR_13, significantly higher than the 58 found in IZTR_24 (Table S2). This robust profile in IZTR_13 is driven by a greater number of genes involved in oxidative stress (36 genes) and osmotic stress (17 genes), compared to 25 and 11 genes, respectively, in IZTR_24. While the specific RAST annotation utilized did not assign subcategories for “acid stress” in either strain, both possess bile hydrolysis genes—a non-negotiable trait for gut survival. IZTR_24 contains two genes for bile hydrolysis, while IZTR_13 possesses one, suggesting that while IZTR_13 is better equipped for general environmental stressors, IZTR_24 may have a slight enzymatic advantage specifically in handling bile salts. This strain-level heterogeneity mirrors recent comparative genomics in *Lactiplantibacillus*, which consistently report wide dispersion of stress tolerance and probiotic marker genes across *L. plantarum* genomes and diversity within the *L. paraplantarum* group [25,26,27,28]. The result confirmed that the presence of specific genes related to strains’ probiotic potential is strain-specific rather than encoded by a single conserved module [29].

From a nutritional perspective, IZTR_13 demonstrates a superior capability for the biosynthesis of vitamins and cofactors, a highly desirable probiotic trait for host health. IZTR_13 contains 142 genes in the cofactors and vitamins category, compared to 130 in IZTR_24. This difference is highlighted in the synthesis of folate and pterines genes, where IZTR_13 possesses 50 genes versus 42 in IZTR_24, and in riboflavin (Vitamin B2) metabolism, where IZTR_13 has 40 genes compared to 38 in IZTR_24. Consequently, IZTR_13 may offer a higher value as a bio-producer of B-vitamins in the gut.

Finally, regarding genetic stability and safety, there is a notable divergence in the presence of mobile genetic elements. IZTR_24 appears to be the more genetically stable strain, containing only 6 genes associated with phages, prophages, transposable elements, and plasmids, whereas IZTR_13 contains 30 such elements. While the higher number of mobile elements can suggest plasticity and adaptation, in a commercial probiotic context, the lower number is often preferred to minimize the risk of horizontal gene transfer. Both strains show similar profiles regarding the absence of genes associated with toxins or virulence factors (according to VFDB) and ARG (according to Megares and CARD databases), supporting their potential safety for probiotic applications.

**Figure 2:**
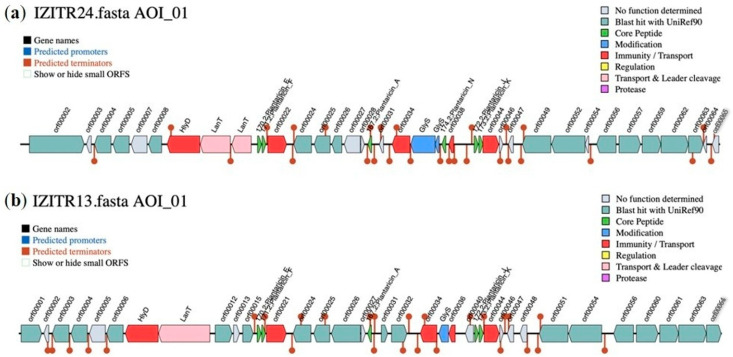
Genomic maps of the clusters of the plantaricins (class IIb bacteriocines) regions of (**a**) *Lactiplantibacillus plantarum* IZITR_24; (**b**) *Lactiplantibacillus paraplantarum* IZITR_13.

**Figure 3:**
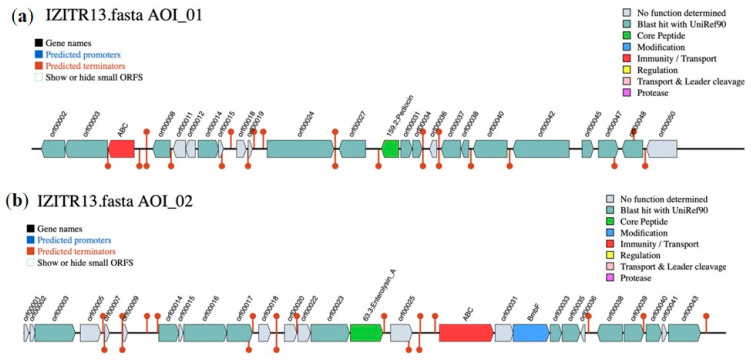
Genomic maps of the *L. paraplantarum* IZITR_13 clusters of (**a**) pediocin (class IIa bacteriocine) region; (**b**) enterolysin_A (cell wall-degrading bacteriocin) region.

Whole genome analysis identified conserved class IIb plantaricin operons in both isolates, with gene pairs plnJK and plnEF detected alongside their cognate transport/immunity modules (Figure 2a,b); in *L. paraplantarum* IZITR_13, this repertoire is augmented by class IIa pediocin (Figure 3a) and the cell wall-degrading bacteriocin enterolysin A loci (Figure 3b). The wide distribution, mosaic organization, and regulatory plasticity of plnEF loci across *L. plantarum* from diverse niches are well documented and align with the observed operon architecture [30,31]. Two-peptide plantaricins act synergistically: high-resolution structures show helix–helix packing via GxxxG/SxxxG motifs, consistent with membrane insertion and pore formation when both peptides are present [32,33].

Beyond membrane permeabilization, target recognition contributes to potency and spectrum; for PlnEF, resistance mapping in *Lactiplantibacillus* pinpointed CorC, a Mg^2+^/Co^2+^ efflux protein, as the receptor [34], while PlnJK sensitivity depends on a predicted amino acid transporter [35], and related two-peptide systems can also hinge on cell wall enzymes such as UppP [36]. These mechanisms were compatible with our neutralized LCFS phenotypes against Gram-positive staphylococci, where peptide-forward activity persisted despite the removal of acid stress. Class IIa pediocins (present in IZITR_13) typically exploit the mannose phosphotransferase system on susceptible Gram-positives and are established anti-staphylococcal agents [37], whereas enterolysin A (also in IZITR_13) is a bacteriolysin that cleaves peptidoglycan and displays pronounced activity against staphylococci [38,39]. Collectively, these genome-encoded effectors rationalize the strong, dose-dependent antibiofilm and growth inhibitory effects that were established for the neutralized LCFS [40,41].

### 2.2. In Vitro Assessment of the Probiotic Potential

**Figure 4:**
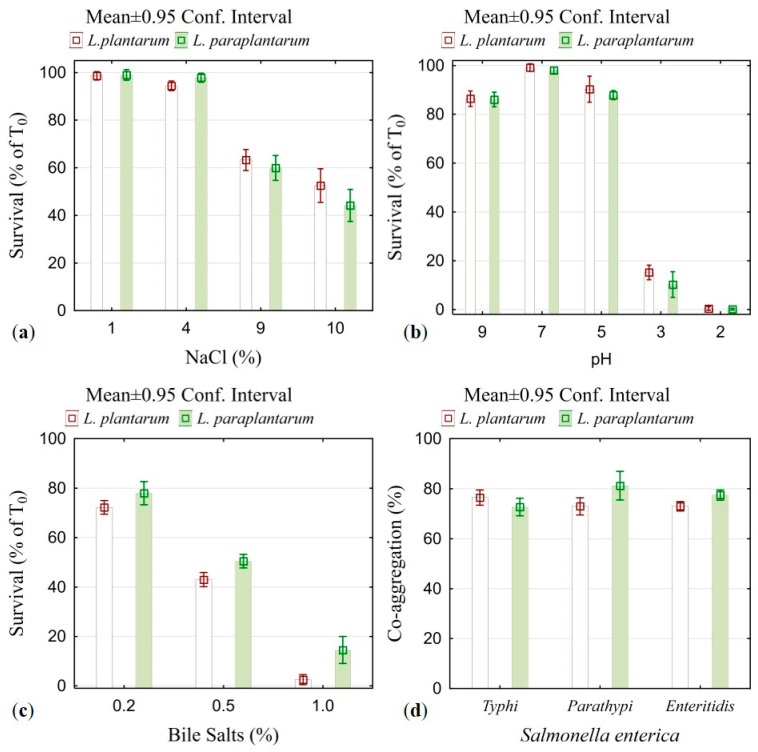
Probiotic potential of *Lactiplantibacillus plantarum* IZITR_24 and *Lactiplantibacillus paraplantarum* IZITR_13: (**a**) Osmotic tolerance (NaCl, *w*/*v* %): survival (%) after exposure to 0–10% NaCl; (**b**) acid tolerance (pH): survival across the tested pH range, compared to the initial cell count (T_0_); (**c**) bile salt tolerance: survival after 2 h exposure to bile salts across the tested concentrations; data are expressed as survival (%) compared to the initial cell count (T_0_); (**d**) co-aggregation with Salmonella enterica (three serotypes): co-aggregation (%) of each producer with the indicated serotypes after the assay period, reported relative to the initial optical density.

The two isolated strains—*Lactiplantibacillus plantarum* IZITR_24 and *Lactiplantibacillus paraplantarum* IZITR_13—were subjected to a comprehensive evaluation to determine their suitability as probiotic candidates according to the current criteria for the selection of probiotic strains with potential for use in food and pharmaceutical products [42,43]. The results of the individual tests are presented in Figure 4.

In the osmotic challenge, both producers maintained >90% survival up to 4% NaCl, with viability dropping at higher salinities; at 10% NaCl, mean survival was 52.5% for *L. plantarum* IZITR_24 and 44.2% for *L. paraplantarum* IZITR_13 (Figure 4a). These data position IZITR_24 as the more osmotolerant strain, yet both isolates remain within a robust performance window typically expected for food-origin *Lactiplantibacillus* under hyperosmotic stress.

This pattern is consistent with recent reports on *L. plantarum*, where many strains grow well at 5% NaCl and some tolerate 8%, supported by genomic evidence for compatible solute transport, membrane remodeling, and general stress regulons [44]. Experimental evidence indicates that *L. plantarum* can endure NaCl stress while undergoing physiological reprogramming (e.g., EPS production, surface characteristics) [45], and that prior adaptation to NaCl can improve aggregation and adhesion—traits significant for probiotic viability [46]. For *L. paraplantarum*, strain-level sensitivity above ~5% NaCl has been observed (e.g., *L. paraplantarum* 11-1 showed growth impairment ≥5%), which aligns with our relatively steeper decline for IZITR_13 at the highest test point [47].

In the 2 h, 37 °C MRS exposure, both producers showed low survival at pH 3.0—about 15% for *L. plantarum* IZITR_24 and ~10% for *L. paraplantarum* IZITR_13—and no recovery at pH 2.0 (Figure 4b). The low acid survival observed for both strains aligns with the lack of annotated, strain-specific acid stress modules in the current RAST/KEGG subcategories, indicating no clear genomic signature for pronounced acid tolerance. Placed against the literature, the result for *L. plantarum* is below many reports in which selected strains retain high viability at pH 3 (often >90%) and, in some cases, endure brief exposure to pH 2 [48,49,50]. For *L. paraplantarum*, published data show heterogeneous performance: some strains remain viable after hours at pH 2.5–3.0 but also lose viability at pH 2.0, which is in line with our observation [51,52]. Taken together, these comparisons support the view that acid tolerance is primarily strain-specific rather than strictly species-defined within *Lactiplantibacillus* [53], while mechanistic studies in *L. plantarum* (e.g., LM1001) explain such divergence through strain-level differences in membrane remodeling and pH homeostasis responses [29].

Tolerance to bile salts is essential for the effectiveness of lactic acid bacteria (LAB) as probiotics, since after passing through the stomach, they are inevitably exposed to bile salts in the small intestine. These salts function as surface-active agents (detergents), compromising cell membranes, which results in bacterial lysis and loss of viability [54]. The conducted analysis indicated that both strains retained substantial short-term viability with no significant difference (ANOVA, *p* > 0.05): at 0.2% bile, survival was ~72% for *L. plantarum* IZITR_24 and ~78% for *L. paraplantarum* IZITR_13 (Figure 4c). The increase in the bile salt concentration led to a significant decline (ANOVA *p* < 0.001) in the cell numbers. The presence of bile hydrolysis genes (two in *L. plantarum* IZITR_24; one in *L. paraplantarum* IZITR_13) matches the moderate short-term bile tolerance and the sharp decline at higher concentrations, consistent with limited but functional bile adaptation. These values are in agreement with the ranges reported for *Lactiplantibacillus plantarum*, where many strains withstand 0.3–0.5% bile for 2–3 h but with marked inter-strain variability [55,56]. For *L. paraplantarum*, published probiotic candidates likewise show bile tolerance around the 0.3% benchmark [52,57,58]. Taken together, our data support the view that bile tolerance in *Lactiplantibacillus* is predominantly strain-dependent rather than species-determined [55,56,59]. Both strains meet widely used screening expectations for initial probiotic robustness [60,61].

Both producers exhibited strong co-aggregation with *S. enterica*. The high co-aggregation percentages are compatible with a robust surface/exopolysaccharide toolkit captured by broad RAST/KEGG categories. Across serotypes, values fell in a narrow, high range (66–78%). With *S. Typhi*, the two strains were indistinguishable (74% each), whereas *L. paraplantarum* IZITR_13 modestly outperformed *L. plantarum* IZITR_24 against *S. Paratyphi* (78% vs. 73%) and *S. Enteritidis* (77% vs. 74%) (Figure 4d). These magnitudes sit at the upper end of the published ranges for lactobacilli: co-aggregation by *Lactiplantibacillus* spp. with *S. enterica* often spans ~20–66% depending on the strain, pairing, and assay time [62,63]. Reports of higher values—including >55% with *S. Typhimurium* for multiple *L. plantarum* isolates —highlight pronounced strain and pathogen specificity consistent with our serotype-dependent pattern [63,64]. In parallel, studies linking lactobacilli surface traits (autoaggregation/hydrophobicity) to co-aggregation and reduced *Salmonella* biofilms further support the functional relevance of the high percentages observed here [65,66].

### 2.3. Antimicrobial Activity of L. plantarum IZITR_24 and L. paraplantarum IZITR_13

#### 2.3.1. Antagonistic Effect of *L. plantarum*_IZITR_24 and *L. paraplantarum*_IZITR_13 Using Plug Diffusion

**Figure 5:**
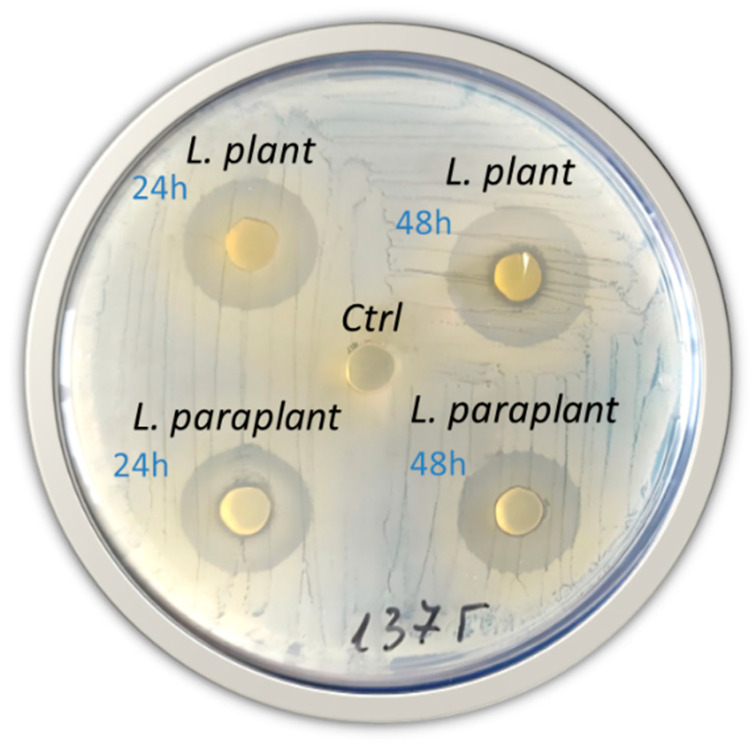
Antagonistic effect of *Lactiplantibacillus plantarum* IZITR_24 and *Lactiplantibacillus paraplantarum* IZITR_13 in an agar plug diffusion assay against *Staphylococcus aureus*. Ctrl—uninoculated sterile MRS agar used as a control.

Across 50 *S. aureus* isolates, agar plug diffusion yielded reproducible halos of 19 ± 8 mm for *L. plantarum* IZITR_24 and 18 ± 6 mm for *L. paraplantarum* IZITR_13 (mean ± 95% CI), with overlapping intervals and representative bactericidal “clear cores” under 48 h producer plugs (vs. lighter, mainly bacteriostatic halos at 24 h); accordingly, 48 h producer cultures were used for downstream LCFS work (Figure 5). These magnitudes and the isolate-to-isolate dispersion fit well within published plug diffusion screens where LAB producers are tested directly against *S. aureus* and MRSA, including food-origin and clinical strains, and where the outcome depends strongly on the producer strain, plug age, and matrix [18,19,67,68,69,70,71,72]. The tendency for larger or “clear-core” halos with 48 h plugs is consistent with late-phase accumulation of postbiotics/bacteriocins (plantaricins) in dense cultures [73], and matches evidence that LAB supernatants can reprogram *S. aureus* virulence circuits (e.g., *agr*/*sae*, *sea*/*tst*/*spa*/*sbi*) and attenuate early biofilm establishment—mechanisms that rationalize strong halo activity even when planktonic MICs are comparatively higher [13,14,22]. Reports of potent anti-staphylococcal plantaricins and neutralized CFS activity further support a multifactorial, peptide-forward basis for our plug diffusion results [17,19,74].

Prior to selecting the working postbiotic, we quantified non-peptidic contributors in the producer supernatants. A GC–MS screen of the cell-free supernatants (CFSs) showed that total free fatty acids (FFAs) constituted < 0.1% of the LCFS mass, with the FFA pool (normalized within the pool) dominated by oleic acid (C18:1; 68.65% in IZITR_24; 57.28% in IZITR_13), followed by linoleic (C18:2; 3.23% vs. 8.53%), palmitic (C16:0; 5.17% vs. 7.51%), and lower fractions of C4:0–C8:0 and C18:0 (Table S3). Because the absolute FFA content was extremely low, and to isolate peptide-driven effects, all downstream assays used pH-neutralized (7.0) LCFS. This choice also controls for organic acids, which in LAB CFSs are typically lactic and acetic (often the predominant soluble acids in *L. plantarum*) [75]. Neutralization allowed assessing antibiofilm/antigrowth activity with minimal acid confounding, consistent with prior CFS studies [22].

#### 2.3.2. Biofilm and Cell Growth Inhibition by LCFS from *L. plantarum* IZITR_24 and *L. paraplantarum* IZITR_13

**Figure 6:**
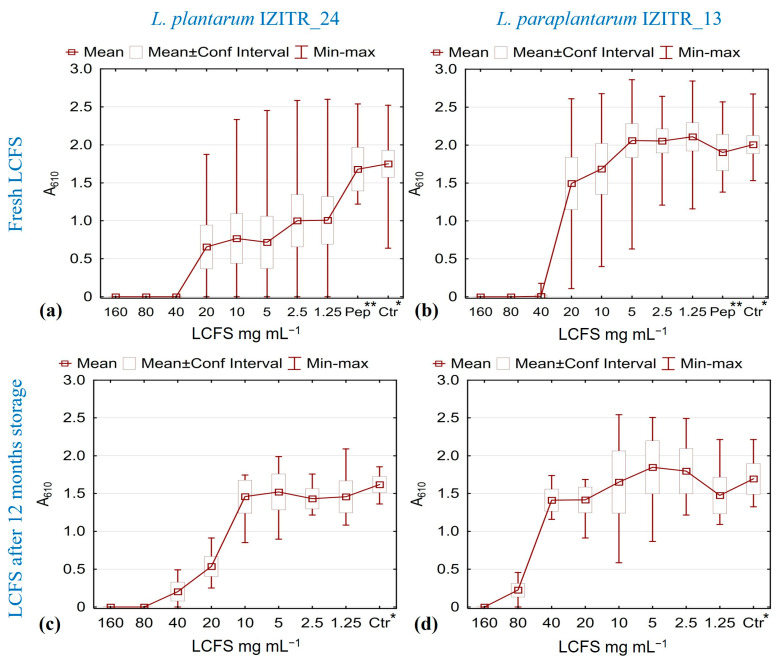
Biofilm inhibitory effect of a lyophilized cell-free culture supernatant (LCFS) from lactic acid bacteria (LAB) against 50 clinical *S. aureus* isolates, applied immediately after lyophilization (24 h) and after 12 months of cold storage of the lyophilizate (2–8 °C): (**a**) *L. plantarum* IZITR_24—24 h; (**b**) *L. paraplantarum* IZITR_13—24 h; (**c**) *L. plantarum* IZITR_24—12 months; (**d**) *L. paraplantarum* IZITR_13—12 months. * Ctr—uninoculated lyophilized medium; ** Pep—pepsin-treated, neutralized LCFS (160 mg mL^−1^).

The neutralized (pH 7.0) LCFSs of both producers suppressed *S. aureus* biofilm formation for all of the tested strains (*n* = 50) in a clear, dose-dependent manner over 1.25–160 mg mL^−1^. In the freshly prepared LCFS, *L. plantarum* IZITR_24 led to a complete biofilm inhibition at 40 mg mL^−1^, with strain-specific responses and detectable effects down to 2.5 mg mL^−1^ in a minority of strains (Figure 6a); *L. paraplantarum* IZITR_13 also reached an MBIC around 40 mg mL^−1^ in the 24 h lyophilizate, but showed no inhibition at concentrations ≤ 20 mg·mL^−1^ (Figure 6b). For stability assessment, the tests were repeated with lyophilized supernatants subjected to 12 months’ cold storage (2–8 °C). This led to a significant reduction (*p* < 0.001) but not to a complete loss of activity. Concentrations of 40 mg·mL^−1^ *L. plantarum* IZITR_24 LCFS inhibited biofilm formation in ~75% of isolates, with full inhibition at 80 mg mL^−1^. A concentration of 160 mg mL^−1^ *L. paraplantarum* IZITR_13 LCFS was required for consistent, near-complete suppression across the panel (Figure 6c,d). The relative ranking was preserved (IZITR_24 > IZITR_13). Assays were run in TSBGSP and compared to lyophilized medium controls to exclude matrix effects. In a protease control, pepsin-treated, neutralized LCFSs lost inhibitory activity, with readouts approaching those of the uninoculated lyophilized medium (Figure 6a,b). This protease susceptibility supports a peptide-driven mode of action for both IZITR_24 and IZITR_13 LCFSs.

The results are consistent with the current literature on the effectiveness of LAB postbiotics against *S. aureus*, as multiple studies demonstrate considerable antibiofilm activity in LAB cell-free supernatants, emphasizing strain-specific potency and temporal influences [18,19,20,76,77]. The neutralization of LCFSs to pH 7.0 suggests additional contributions beyond acidification, consistent with findings that LAB CFSs can down-regulate *S. aureus* virulence genes (e.g., *agr*/*sae*, *sea*/*tst*/*spa*/*sbi*) in growth-permissive conditions [22]. Recent studies indicate that LAB-CFS adversely affects essential biofilm characteristics, including surface hydrophobicity, eDNA/PIA, and motility proxies, while also decreasing biomass in a dose responsive manner [13]. Additionally, *L. rhamnosus* GG CFS has been shown to inhibit both mono- and dual-species biofilms without promoting resistance [14]. Our genomic analysis indicates that class IIb plantaricin pairs plnJK/plnEF are present in both producers, alongside pediocin and enterolysin A in IZITR_13. These data support a peptide-forward mechanism for activity. Recent studies on bacteriocins reveal that plantaricins exhibit anti-MRSA properties at low concentrations [17], which aligns with the pronounced antibiofilm effect of IZITR_24 LCFS and the maintained, albeit reduced, activity following storage.

At 40 mg mL^−1^, the LCFS of *L. plantarum* showed no effect on planktonic growth. Across the isolate panel, the MIC was between 80 and 160 mg mL^−1^; to classify the mode of action, cultures were taken from the MIC well and the next higher dilutions. The MIC proved bacteriostatic, while at higher concentrations, a bactericidal, strain-specific effect was observed. For *L. paraplantarum*, the concentrations that inhibit biofilm formation did not inhibit planktonic growth; bacteriostasis appeared only at the upper end of the tested range (80–160 mg mL^−1^), and no bactericidal effect was detected. Based on these data, LD_99_ and MBI_99_ values with 95% confidence intervals were calculated by probability analysis (PROBIT) and are reported in Table 1.

Overall, the planktonic suppression requires higher doses for both LCFSs—an MBIC < MIC separation that is widely reported for LAB cell-free supernatants against *S. aureus* and reflects strain- and timing-dependent effects [13,18,19,20,74]. The pH-neutralized preparations retaining activity, together with negligible fatty acid content, and the loss of activity after pepsin treatment for both IZITR_13 and IZITR_24 LCFS argue for a peptide-forward mechanism consistent with bacteriocin (plantaricin) activity documented against *S. aureus* [17,22,73,74].

### 2.4. Biofilm Visualization

#### 2.4.1. Confocal Laser Scanning Microscopy (CLSM)

**Figure 7:**
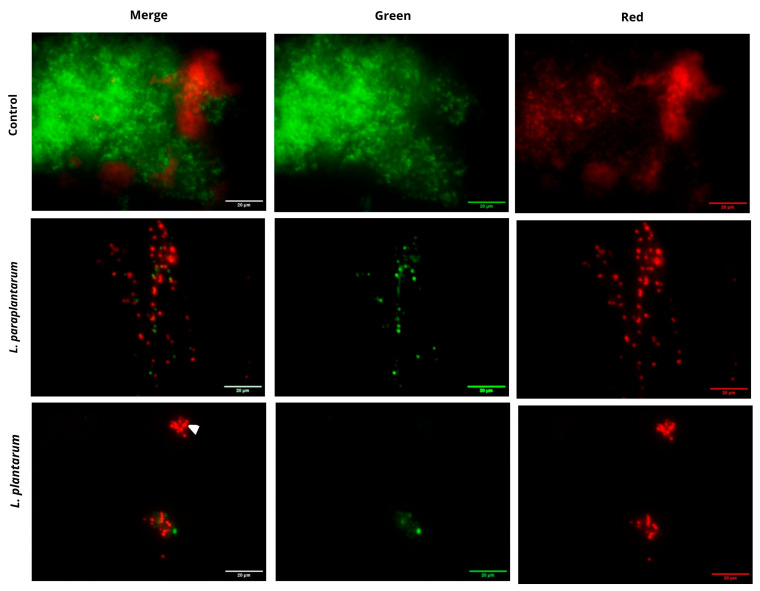
Determination of the viability of *S. aureus* cells within the biofilm cultivated in the presence of LCFS from *Lactiplantibacillus plantarum* IZITR_24 and *Lactiplantibacillus paraplantarum*_IZITR_13 at concentrations 25% MBIC. Bars = 20 μm. white triangle—cells with enlarged dimensions.

To monitor the effect of treatment with different supernatants on the viability of bacterial cells within the biofilm, we performed fluorescent staining, where all viable cells fluoresced green and all non-viable cells fluoresced red. Observation of the control sample revealed a well-developed biofilm with a predominance of live, metabolically active cells and a smaller distribution of dead cells, which is a normal process during biofilm maturation (Figure 7). Upon application of the supernatants from *L. plantarum* and *L. paraplantarum* at a concentration of 25% MBIC, we observed a reduction in biofilm biomass. Compared with the control, where the overlay of the fluorescent signal demonstrated a multilayered biofilm structure with vertical growth, under the influence of LCFSs from both strains, cells were more sparsely distributed, forming small aggregates consisting predominantly of non-viable (red-stained) cells. Across all treated samples, the biofilms appeared monolayered, and enlarged cells (white triangle) were also detected. Fluorescence analysis demonstrated that treatment with the tested supernatants resulted in the exfoliation of biofilm mass and reduced bacterial cell viability within the biofilms.

The observed effects are supported by numerous other studies investigating the application of products derived from the family Lactobacillaceae against *Staphylococcus* biofilms. Mohapatra et al. [78] demonstrated that low-molecular-weight products of *Lactiplantibacillus plantarum* SJ33 inhibited and disrupted biofilms formed by *S. aureus* MTCC96 and methicillin-resistant *S. aureus* (MRSA). A year later, Mao et al. [13] reported that cell-free supernatants from lactobacilli inhibited the biofilm formation of wild-type *S. aureus* and affected different metabolic pathways. Nowak-Lange et al. [11] reported that probiotics synthesized by *Lactiplantibacillus pentosus* B1 inhibited and eradicated the biofilms of *S. aureus*, *Escherichia coli*, *Streptococcus pyogenes*, and *Cutibacterium acnes*. There is information that cell-free supernatants from lactobacilli, isolated from human breast milk and infant fecal samples, affected the growth and spatial conformation of biofilms formed by *S. aureus* and *Pseudomonas aeruginosa* [79].

#### 2.4.2. Scanning Electron Microscopy (SEM)

**Figure 8:**
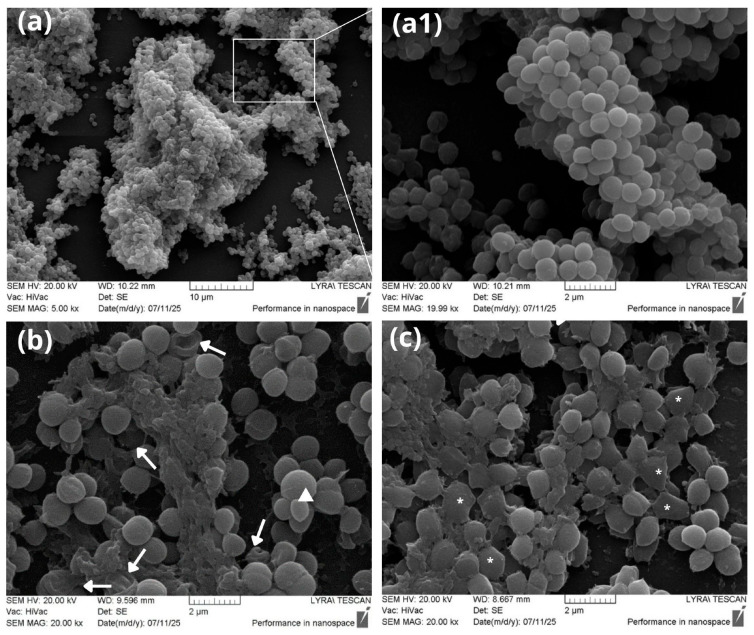
Scanning electron microscopy of *S. aureus* biofilms cultivated in the presence of (**a**) control—untreated sample; (**a1**) enlarged view of a selected field from the control sample; (**b**) supernatant from *Lactiplantibacillus plantarum* IZITR_24 at 25% MBIC concentration; (**c**) supernatant from *Lactiplantibacillus paraplantarum* IZITR_13 at 25% MBIC concentration; white arrow—cell invaginations; white star—cells with abnormal shape; white triangle—cells with enlarged dimensions. Bars = 2 μm.

To investigate the 3D structure of biofilms and the occurrence of potential morphological aberrations, *S. aureus* was cultured in the presence of LCFS from *L. plantarum* IZITR_24 and *L. paraplantarum*_IZITR_13 again in a concentration of 25% of MBIC, followed by preparation for scanning electron microscopy. Observation of the 48 h biofilms in comparison with the control sample revealed the presence of a well-developed and structured biofilm (Figure 8a,a1). Multilayered cellular consortia were observed, exhibiting the species-specific three-dimensional “mushroom-like” structure and a series of visible openings in the interstitial cavities. Such typical biofilm morphology has been repeatedly described in the scientific literature [53,79,80,81]. At the morphological level, it can be concluded that in the control sample, cells with a spherical shape and dimensions typical for the species, approximately 0.5–1.0 μm, were observed. They exhibited a smooth surface, clearly defined boundaries, and were captured in a dividing form (Figure 8a1). In contrast, despite exposure only to low, sub-inhibitory concentrations (25% of MBIC) of both LCFS preparations, the treated *S. aureus* strains displayed significant morphological alterations. In the biofilms cultivated in the presence of the *L. plantarum* supernatant, damaged cells with significant invaginations were observed, including radial indentations along the length of the cells with presumed leakage of cellular contents (Figure 8b—white arrows). Occurrence of an abnormally large cell was also recorded (white triangle). Similar changes have been reported by other research groups, for example, upon treatment with berberine, which affects the membrane lipid structure [82], serrawett in W2-FL10, which freely permeates bacterial membranes [83], bacteriocins from *L. plantarum* [23], as well as under the influence of cell-free supernatants from the same species [84]. The samples cultivated in the presence of the *L. paraplantarum* supernatant contained atypical cells that, instead of a spherical form, displayed flattened and polygonal shapes (Figure 8c—white stars). Morphological disturbances of this type are processes observed under the influence of various antibacterial agents, such as bacteriocins synthesized by *L. salivarius* [85], microbots associated with and delivering antimicrobial peptides [86], amphiphilic cationic coumarin derivatives, and others [87]. Comparing the biofilm architecture in the control sample with that in the treated samples, we can conclude that the treated samples exhibited thinner, single- or double-layered biofilms lacking multilayered complexity.

## 3. Materials and Methods

### 3.1. Bacterial Strains and Culture Conditions

#### 3.1.1. Producer Strains: Origin, Identification, and Maintenance

Two food-origin LAB were used as postbiotic producers: *Lactiplantibacillus plantarum* IZITR_24 and *Lactiplantibacillus paraplantarum* IZITR_13, which were obtained from one batch of traditionally prepared Bulgarian mixed-vegetable pickles (turshiya), comprising cauliflower, carrots, peppers, and green tomatoes. Fermentation proceeded spontaneously from the autochthonous vegetable microbiota (no commercial starter), prepared in household conditions (Plovdiv Province, Bulgaria) without imposed anaerobiosis at 10–15 °C. LAB were isolated and initially identified through standard laboratory protocols [88]. The brine from the fermented vegetables (turshiya) was subjected to ten-fold serial dilutions in sterile 0.9% NaCl. An amount of 100 µL from each dilution was spread-plated on De Man, Rogosa, Sharpe (MRS) agar, and the plates were incubated in an anaerobic jar (Merck, Darmstadt, Germany) at 30 °C for 48 h. Single distinct colonies that developed on the MRS agar were selected for further analysis and purified by streaking them onto the new MRS agar plates. Bacterial isolates were initially identified based on colony morphology, culture characteristics, microscopic appearance, Gram staining, and catalase production, as stated in Bergey’s Manual of Systematic Bacteriology [89]. Organisms classified as Gram-positive, non-spore-forming, and catalase-negative rods were tentatively identified as *Lactobacillus* spp. For medium-term maintenance, the isolated strains were stored as frozen working stocks at −20 °C in sterile 10% (*w*/*v*) skim milk (Merck, Darmstadt, Germany) and were revived on MRS prior to each experiment. Both strains were subjected to DNA extraction, and whole genome sequencing.

#### 3.1.2. *Staphylococcus aureus*

The strains (*n* = 50) used for the inhibitory tests were obtained from the Microorganisms Culture Collection of the Department of Biochemistry and Microbiology (Paisii Hilendarski Plovdiv University, Plovdiv, Bulgaria). The strains were cultured in tryptic soy broth (TSB, Merck, Darmstadt, Germany) supplemented with 1% (*w*/*v*) glucose and 2% (*w*/*v*) NaCl [18]. *S. aureus* inoculums were standardized by homogenization in sterile 0.85% NaCl, and all suspensions were diluted to 1.5 × 108 CFU mL^−1^ using a McFarland densitometer DEN-1 (Grant-bio, Cambridge, UK) prior to inoculation [90].

### 3.2. DNA Extraction, Sequencing, and Assembly

Genomic DNA was isolated from IZTR_13 and IZTR_24 using the QIAamp DNA Microbiome Kit (QIAGEN, Hilden, Germany). DNA quantity and integrity were assessed with a Qubit 4 fluorometer (Thermo Fisher Scientific, Waltham, MA, USA) and by agarose gel electrophoresis. Long-read library preparation for Oxford Nanopore sequencing was performed with the Ligation Sequencing Kit SQK-RBK114 (Oxford Nanopore Technologies, Oxford, UK) following the manufacturer’s instructions, using 200 ng of total DNA. The resulting libraries were sequenced on a MinION platform equipped with an R10 flow cell (Oxford Nanopore Technologies, Oxford, UK). Guppy v6.5.7 (Oxford Nanopore Technologies, Oxford, UK) was used offline for base calling and quality control. Adapter trimming was carried out with Porechop v0.2.4 (https://github.com/rrwick/Porechop, accessed on 15 December 2025) using default parameters. Genome assembly was performed de novo with Flye v2.9.2, excluding reads shorter than 1 kb. The draft assembly was refined using Racon v1.4.21 (https://github.com/isovic/racon, accessed on 15 December 2025) followed by Medaka v1.8.1 (https://github.com/nanoporetech/medaka, accessed on 15 December 2025). Assembly quality metrics were obtained with CheckM v1.1.6. Visualization of the circular chromosome was generated using Proksee (https://proksee.ca/, accessed on 15 December 2025). The complete genome sequences are submitted in GenBank under the BioProject PRJNA1113526, Accession number IZITR_13 (Organism CP156752) and Accession number IZITR_24 (Organism CP156759).

### 3.3. Genome-Based Identification and Multilocus Sequence Typing (MLST)

MLST profiling was conducted with the PubMLST platform (https://pubmlst.org, accessed on 15 December 2025). In addition, whole genome phylogeny was inferred using the Type (Strain) Genome Server (TYGS; https://tygs.dsmz.de/, accessed on 15 December 2025).

### 3.4. Genome Annotation

The assembled IZITR_13 and IZITR_24 genomes were submitted to NCBI for automated annotation via the Prokaryotic Genome Annotation Pipeline (PGAP), which also provided the accession number. The resulting GenBank file was subsequently re-annotated using the RAST server. Functional gene assignments were complemented with KEGG resources and BlastKOALA, applied to predicted protein sequences obtained from the PGAP output. Genes associated with probiotic functions were manually curated using both RAST and KEGG annotations. Screening for AMR determinants and virulence factors (VFs) was conducted with Abricate (https://github.com/tseemann/abricate, accessed on 15 December 2025) using default parameters against CARD, MEGARes, and VFDB. Potential bacteriocin gene clusters were predicted using BAGEL4 and AntiSMASH 7.0 (https://antismash.secondarymetabolites.org/, accessed on 15 December 2025).

### 3.5. Probiotic Potential Assessment

#### 3.5.1. Cultures and Inoculum Standardization

Overnight cultures of *L. plantarum* IZITR_24 and *L. paraplantarum* IZITR_13 were grown in MRS at 30 °C, harvested (5000× *g*, 10 min), washed twice in sterile 0.85% NaCl, and resuspended in fresh MRS. Inocula were standardized to 0.5 McFarland (1.5 × 10^8^ CFU mL^−1^) from which time-zero counts (T_0_) and end-time counts (T) were determined by serial dilution and spread plating in triplicates on MRS agar for each individual test [91]. Survival (%) for each assay was calculated as (CFUₜ/CFU_0_) × 100.

#### 3.5.2. Osmotic Stress (NaCl)

Aliquots of 1.0 mL standardized cell suspension (McF = 0.5) were incubated for 2 h at 37 °C (static) in MRS adjusted to 0–10% (*w*/*v*) NaCl. Post-exposure, cells were immediately diluted in 0.85% NaCl and plated on MRS agar; survival (%) was calculated against T_0_. Results were interpreted as the percent viability retained at each salinity and used to compare strain performance across the gradient [44].

#### 3.5.3. Acid Tolerance (pH)

Aliquots of 1.0 mL standardized cell suspension (McF = 0.5) were incubated for 2 h at 37 °C in MRS adjusted with HCl to pH 2.0, pH 3.0, pH 5.0, pH 7.0, and pH 9.0. Following exposure, cells were washed (0.85% NaCl), plated on MRS agar, and survival (%) was calculated vs. T_0_. Outcomes were interpreted as retention of culturability at each pH set-point [92].

#### 3.5.4. Bile Salt Tolerance

Aliquots of 1 mL were exposed for 2 h at 37 °C in MRS containing bile salts (e.g., Oxgall) at 0.2%, 0.5%, and 1% concentrations. Cells were recovered by dilution/plating on MRS agar and survival (%) computed relative to T_0_. Results were interpreted as short-term bile survival at physiologically relevant levels (0.2–1%, *w*/*v*) and used to compare strains across the concentration series [92].

#### 3.5.5. Co-Aggregation with Pathogens

Co-aggregation was assessed against three *Salmonella enterica* subsp. *enterica* serotypes (*Typhi*, *Paratyphi*, and *Enteritidis*). Equal volumes of 0.1 mL LAB and pathogen suspensions, each set at 0.5 McF (1.5 × 10^8^ CFU mL^−1^) in PBS, were mixed in flat-bottomed 96-well polystyrene microtiter plates and incubated at 37 °C (static). After 1 h, OD_620_ of all wells was read on a Multiskan FC spectrophotometer (Thermo Scientific, Shanghai, China). Co-aggregation (%) was calculated from optical densities using the Collado/Handley formula:

$$\%\;CoAgg=\frac{(A_{LAB}+A_{path})/2-A_{mix}}{(A_{LAB}+A_{path})/2}\times 100$$

where *A_LAB_* and *A_path_* are the OD values of monocultures and *A_mix_* is the OD of the mixture at the readout time. Replicates (eight per experiment) were averaged and reported as mean ± 95% CI [66].

### 3.6. Preparation of Cell-Free Culture Supernatants (CFSs) and Lyophilized CFSs (LCFSs)

MRS broth (200 mL) was inoculated with 1% inoculum and producer strains were incubated anaerobically without shaking at 30 °C for 48 h [93]. To obtain cell-free supernatants (CFSs), the bacterial cells were pelleted by centrifugation (10,000× *g*, 20 min, 4 °C), and the supernatants were sterile-filtered (0.22 µm). The sterile CFSs and pure MRS medium were frozen at −80 °C and lyophilized (Labcocno FreeZone 4.5; Labconco Corporation, Kansas City, MI, USA). The lyophilized CFSs (LCFSs) were weighted and stored at −20 °C [72], and reconstituted in sterile deionized water immediately before use.

### 3.7. Determination of the Fatty Acid Composition of the Lipid Fraction Isolated from Lyophilized Supernatant

The fatty acid composition of lipids isolated from lyophilized microbial biomass was determined by gas chromatography after transesterification of the sample with 2% (*v*/*v*) H_2_SO_4_ in CH_3_OH at 50 °C as described by Christie [94]. The resulting fatty acid esters were purified by thin-layer chromatography on silica gel plates measuring 20 cm × 20 cm, coated with a 0.2 mm layer of Silica Gel 60 G (Merck Group, St. Louis, MO, USA) with a mobile phase of n-hexane:acetone in a ratio of 100:8. Gas chromatography analysis was performed on a Thermo Fisher Scientific chromatograph, model TRACE 1610 GC (Waltham, MA, USA), autosampler model AI 1310, FID detector, equipped with a Thermo Fisher Scientific column, TR-FAME, length 100 m, ID 0.25 mm, film 0.20 µm. Carrier gas—nitrogen, with a constant flow of 1 mL/min. Split mode—split, injector temperature 240 °C, FID detector temperature 260 °C, temperature program: target value 100 °C, hold time 0.2 °C/min; from 100 °C to 240 °C at a heating rate of 2 °C/min and holding at 240 °C for 25 min. The quantity of the detected components was determined using Chromeleon 7 software by a processing method based on the peak area of the GC/FID chromatogram and internal standardization, and their identification was based on the retention times with those of a standard mixture of fatty acid methyl esters (FAME MIX Supelco, Merck Group, St. Louis, MO, USA), chromatographed under the same conditions.

### 3.8. Antagonistic Activity Screening

Preliminary antagonism screening used an agar plug diffusion method: Fresh (24 h) cultures of *L. plantarum* and *L. paraplantarum* were spread-plated on MRS and incubated in anaerobic jars (Merck, Darmstadt, Germany) at 30 °C for 24 and 48 h. Plugs with 6 mm diameter were aseptically cut and transferred onto MHA (HiMedia, Mumbai, India) previously lawned with *S. aureus* (1.5 × 10^8^ CFU mL^−1^). Following 24 h incubation at 37 °C, the antagonism was determined by the presence/absence of inhibition zones around the agar plugs.

### 3.9. Minimum Inhibitory Concentration (MIC) Against Planktonic Growth

MICs of reconstituted, neutralized (pH 7.0) LCFSs were determined by broth microdilution in 96-well plates [95]. Two-fold dilution series were prepared in 100 µL double-strength tryptic soy broth supplemented with % (*w*/*v*) glucose, 2% (*w*/*v*) NaCl, and 3% (*v*/*v*) human plasma (TSBGSP), as described by Iliev et al. [72]. The final LCFS concentrations range was 160 mg mL^−1^ to 1.25 mg mL^−1^ per well. Wells were inoculated with *S. aureus* to 5 × 10^5^ CFU mL^−1^ and incubated 24 h at 37 °C. OD_620_ was read (Multiskan FC, Thermo Scientific, Shanghai, China), corrected to sterile controls, and MIC recorded as the lowest concentration with no visible growth.

### 3.10. Minimal Biofilm Inhibitory Concentration (MBIC)

*S. aureus* biofilms were grown statically for 24 h at 37 °C in tissue culture 96-well plates using a standardized inoculum (5 × 10^5^ CFU·mL^−1^) in TSBGSP. LCFS dose–responses used eight two-fold dilutions (160 to 1.25 mg·mL^−1^). To verify the peptide-driven nature of the LCFS effects, an additional test was conducted with 160 mg mL^−1^ LCFS (for both IZITR_13 and IZITR_24) neutralized to pH 7.0, treated for 2 h with pepsin (1 mg·mL^−1^), followed by inactivation at 80 °C for 10 min [85]. After incubation, wells were washed (0.85% NaCl), stained with 0.1% crystal violet (10 min), rinsed, and bound dye solubilized in 70% ethanol; absorbance at 620 nm was measured. OD of the sterile control (ODc) and test wells (ODt) were recorded. The MBIC was defined operationally and modeled (see “Statistics”). All isolates used in the panel were pre-verified as strong biofilm producers (ODt/ODc > 4), as described by Stepanović et al. [96], under these conditions.

All MIC/MBIC measurements were performed in technical triplicates in at least two independent experiments for each strain of *S. aureus*. Negative controls included sterile TSBGSP media; for positive controls, *S. aureus* were inoculated in TSBGSP only.

### 3.11. Biofilm Visualization

#### 3.11.1. Confocal Laser Scanning Microscopy (CLSM) of Biofilm Viability

To assess the effect of the tested LCFSs on the viability of *S. aureus* biofilms, we performed fluorescent staining followed by observation with a confocal laser scanning microscope under epifluorescence mode. S. aureus 450G strain was chosen as a strong biofilm producer. It was cultured for 24 h at 37 °C in 24-well plates in the presence of 25% of the MBIC of LCFSs from *L. plantarum* IZITR_24/*L. paraplantarum* IZITR_13 on sterile borosilicate glass coverslips. After treatment, the coverslips with biofilm were washed with PBS to remove non-adherent cells and stained with the Live/Dead BacLight Bacterial Viability Kit (Invitrogen, Carlsbad, CA, USA), according to the manufacturer’s instructions. Subsequently, the samples were mounted onto microscope slides using Fluoromount Mounting Medium (Sigma, New York, NY, USA). Observations were carried out on a Nikon Eclipse Ti-U confocal laser scanning microscope equipped with a 60× Plan Apo objective lens. Images were acquired using a Nikon DS-Fi1 CCD Camera (Melville, NY, USA) and processed with the NIS-Elements software (Ver. 4.0), which enables visualization and overlay of images obtained from different fluorophores. Further image processing was performed using the Icy Bioimaging program (Ver. GPLv3).

#### 3.11.2. Scanning Electron Microscopy (SEM) of Biofilm Architecture

Cultivation experiments were performed with *Staphylococcus aureus* G450 in the presence of 25% of the MBIC of LCFSs from *L. plantarum* IZITR_24/*L. paraplantarum* IZITR_13. Biofilm formation by *S. aureus* was carried out in 24-well plates on sterile polystyrene pieces that had been pretreated with 96% ethanol and UV-sterilized. Following the 24 h at 37 °C incubation period, biofilms were gently washed with PBS and fixed for 2 h with 4% glutaraldehyde in 0.1 M sodium cacodylate buffer (pH 7.2). Samples were subsequently rinsed three times in 0.1 M cacodylate buffer and post-fixed with 1% osmium tetroxide (OsO_4_) in 0.1 M cacodylate buffer for 1 h. Dehydration was performed through a graded ethanol series, after which the samples were mounted on metallic stubs and sputter-coated with gold using an Eduards Sputter Coater. Microscopic observations were performed with a Lyra Tescan scanning electron microscope (Tescan Analytics, Brno, Czech Republic) at an accelerating voltage of 20 kV.

### 3.12. Statistics

Dose–response data from MIC and biofilm assays were analyzed by probit regression to estimate central effects and 95% confidence intervals for MIC and MBIC (SPSS Statistics V26, IBM corp., Armonk, NY, USA). Group comparisons (e.g., media effects, storage stability) used standard parametric/non-parametric tests as appropriate after data screening; significance was set at α = 0.05 (Statistica V12, StatSoft Inc., Tulsa, OK, USA).

## 4. Conclusions

This study establishes *Lactiplantibacillus plantarum* IZITR_24 and *L. paraplantarum* IZITR_13, isolated from traditional Bulgarian turshiyas, as potent reservoirs for anti-staphylococcal defense. Neutralized, lyophilized cell-free supernatants (LCFSs) from IZITR_24 and IZITR_13 successfully inhibited *Staphylococcus aureus* biofilm formation, with IZITR_24 consistently more potent. Across the clinical panel, LCFSs produced clear, dose-dependent biomass reductions, while growth suppression was largely bacteriostatic and occurred at higher doses than antibiofilm effects. Confocal and SEM imaging showed that the exposure to a low sub-inhibitory concentration (25% of MBIC) leads to biofilm thinning, and the formation of only patchy mono-/bilayers with altered cell surface morphology, in line with the quantitative trends. Inhibition persisted after pH neutralization and was abolished by pepsin, and free fatty acids were <0.1% of the preparations—together supporting a proteinaceous (bacteriocin-driven) mechanism. Whole genome sequencing corroborated this interpretation by revealing plantaricin systems (plnJK/plnEF) in both producers (with pediocin and enterolysin A additionally in IZITR_13), yielding a coherent genome-to-phenotype link. The WGS-first approach proved highly effective for this class of work: it prioritizes high-value producers, saves substantial time and resources otherwise spent on broad in vitro screening, and mitigates the poor cross-study comparability caused by variable experimental conditions. Overall, the combined in vitro efficacy and in silico repertoire position these strains not merely as potential probiotic food isolates, but as promising bio-factories for next-generation antimicrobials. Harnessing these nature-derived products presents a promising strategy to disarm *S. aureus* and complement existing therapies.

## Figures and Tables

**Table 1:** Mean value and 95% confidence intervals for minimal biofilm inhibitory (MBIC) and minimal inhibitory (MIC) concentrations (mg·mL^−1^) for the 48 h *L. plantarum* IZITR_24 and *L. paraplantarum* IZITR_13 LCFSs against nasal *S. aureus* strains.

	*L. plantarum* IZITR_24				*L. paraplantarum* IZITR_13			
	MBIC (mg mL^−1^)		MIC (mg mL^−1^)		MBIC (mg mL^−1^)		MIC (mg mL^−1^)	
	LD_50_	LD_99_	LD_50_	LD_99_	LD_50_	LD_99_	LD_50_	LD_99_
Estimate probability	11.590	45.520	57.60	132.00	24.420	53.850	93.47	233.00
Lower bound	7.080	36.610	45.66	95.85	19.380	38.540	83.29	187.20
Upper bound	19.460	64.240	76.85	313.38	31.830	139.380	105.11	333.86

## Data Availability

Data are contained within the article and Supplementary Materials.

## Supplementary Materials

The following supporting information can be downloaded at: https://www.mdpi.com/article/10.3390/ijms27020760/s1.

## Abbreviations

The following abbreviations are used in this manuscript:

CFS	Cell-free supernatant
LCFS	Lyophilized cell-free supernatant
MSSA	Methicillin-sensitive *Staphylococcus aureus*
MRSA	Methicillin-resistant *Staphylococcus aureus*
