# Supplementary material for: Potential of Fermented Food-Derived Lactiplantibacillus Cell-Free Supernatants to Control Staphylococcus aureus Growth and Biofilm Development
Source: Int J Mol Sci. 2026 Jan 12;27(2):760. doi: 10.3390/ijms27020760 (PMC12841026; doi:10.3390/ijms27020760)
Supplement: Supplementary file 1 [file ijms-27-00760-s001.zip › Table S3.docx]

| **Fatty acid** | ***L. plantarum*** | ***L. paraplantarum*** |
| --- | --- | --- |
| Butyric acid C_4:0_ | 0.31 | 1.87 |
| Caprolic acid C_6:0_ | 0.5 | 2.04 |
| Caprilic acid C_8:0_ | 2.69 | 1.02 |
| Myristic acid C_14:0_ | 0.48 | n.d |
| Palmitic acid C_16:0_ | 5.17 | 7.51 |
| Stearic acid C_18:0_ | 2.13 | 3.36 |
| Oleic acid C_18:1_ | 68.65 | 57.28 |
| Linoleic acid C_18:2_ | 3.23 | 8.53 |
| Arachidic acid C_20:0_ | 0.24 | n.d |
